# Profiles of physical activity biographies in relation to life and aging satisfaction in older adults: longitudinal findings

**DOI:** 10.1186/s11556-019-0221-6

**Published:** 2019-08-09

**Authors:** Paul Gellert, Julian Wienert, Jochen P. Ziegelmann, Adelheid Kuhlmey

**Affiliations:** 1 Charité – Universitätsmedizin Berlin, Institute for Medical Sociology and Rehabilitation Science, Virchowweg 22, 10117 Berlin, Germany; 2 Department of Psychology & Methods, Jacobs University Bremen, Res IV, Campus Ring 1, 28759 Bremen, Germany; 3 Division of Health Psychology, Freie Universität Berlin, Habelschwerdter Allee 45, 14195 Berlin, Germany

**Keywords:** Lifespan, Exercise, History, Wellbeing, Quality of life

## Abstract

**Background:**

While there is substantial evidence on the relationship between life satisfaction and present physical activity (PA), less is known about which specific PA biographies are associated with a high quality of life and aging satisfaction. Our objective was to identify classes of PA biographies that may be associated with life and aging satisfaction.

**Methods:**

In this longitudinal study, PA biographies were assessed retrospectively as a baseline, followed by assessments of life and aging satisfaction at six and twelve months in 418 adults aged 60–95. Subgroups with different PA biographies were identified using latent class analysis.

**Results:**

Four distinct PA biographies emerged: increasingly active (35%; *n* = 147); consistently active (25%; *n* = 103); consistently inactive (18%; *n* = 75); and decreasingly active (22%; *n* = 94). Being consistently active was related to life satisfaction (β = .17) and consistent inactivity was associated with aging dissatisfaction (β = −.20) when accounting for current PA levels.

**Conclusions:**

In addition to current PA, our findings emphasize the value of PA biographies for life and aging satisfaction, which could inform lifespan theories of PA and health promotion.

## Introduction

Physical activity (PA) levels, on average, tend to decrease over the course of life. The percentage of adults aged 65 and up that meet physician recommendations for PA is low, ranging from 27 to 44% across surveys [1]. PA is closely related to indicators of successful aging such as avoiding diseases and disability, maintaining mental health, and engaging in an active lifestyle [2–5]. While starting to be more active in old age still has benefits for health, evidence suggests that lifelong participation in PA has an additional positive impact on health [6].

Although most existing literature focuses on average PA levels that decrease with advancing age, there are considerable individual differences between lifespan trajectories of PA biographies [6]. Some older adults remain active, others become active after retirement, and yet others become more sedentary as they age [1, 7]. Alongside longitudinal cohort studies, retrospective interview techniques ask participants to review their PA over the lifespan. A study that used a retrospective interview procedure to measure involvement in PA over the lifespan of master athletes and sedentary older adults showed that the two groups had significantly different trajectories [7]. Athletes continued to have a high level of activity, whereas sedentary adults decreased their activity levels consistently. Further, this study showed that an athletic PA biography was positively related to current PA levels. In a prospective study among older adults, retrospectively-reported PA biography was found to be related to current PA over 8 years [8]. In men and women, self-reported PA biographies from 10 to 19 years of age were significantly associated with current PA in old age. Different life course patterns beyond gender and having an athletic background are not yet investigated.

PA levels are substantially related to life satisfaction [9]. Furthermore, evidence suggests that current PA levels are connected to more positive views on aging and aging satisfaction [10]; however, whether there are is a positive relationship between the individual PA biography and life and aging satisfaction indicators beyond current PA levels is not well understood.

Research investigating the associations between different life course trajectories of older adults and life and aging satisfaction is limited. This knowledge may be valuable for intervention developers and policy makers as strategies could be tailored to groups of older adults with different PA biographies. Thus, this study aimed to identify different classes of PA biographies of older adults based on retrospective self-reporting in order to further understand if an active lifestyle relates with life and aging satisfaction. We assume that the identified PA biography classes are differentially related to indicators of life and aging satisfaction even when accounting for current PA levels with more active profiles are hypothesised to be linked with higher levels of life and aging satisfaction.

## Methods

### Participants and procedure

The present study focused on retrospectively reviewed PA biographies across the lifespan of older adults. The full study design is described elsewhere [11]. Ethical guidelines were followed and clearance from the ethics committee of the German Psychological Society (JZ062009). Participant inclusion criteria included being above the age of 60 and not having a medical inability to perform physical exercise (assessed in an open format with self-reporting). Participants were recruited via newspaper announcements. After they sent back the informed consent form, participants received questionnaires in the mail at baseline (i.e., PA biography), 6 months (i.e., current PA), and again at 12 months (i.e., indicators of life and aging satisfaction). Data collection took place in 2010 across Germany, with most participants coming from Berlin and the surrounding area. At baseline, 418 participants completed the questionnaire. The second questionnaire was completed by 340 participants (81% of baseline) and 335 participants completed the third questionnaire (80% of baseline).

### Measures

The *PA biography* assessment was adapted from the 2005 study “Tracing the development of athletes using retrospective interview methods” [12] and was assessed with four single items: “In your childhood and adolescence, how frequently were you consistently physically active?”; “In your 30s, how frequently were you consistently physically active?”; “In your 50s, how frequently were you consistently physically active?”; and “In your 60s, how frequently were you consistently physically active?” A 6-point Likert-type response format (never, seldom, sometimes, quite often, mostly, and always) was used.

*Current PA* was assessed using two items adapted from a brief assessment of the Longitudinal Aging Study Amsterdam [13]: “In a normal week, how often do you perform physical exercise?” and “In the last four weeks, how often have you performed physical exercise?” Internal consistency was high with an item correlation of .85, *p* < .001 in the present study.

*Life and aging satisfaction* served as the outcome in this study and was assessed using the 17-item Philadelphia Geriatric Center Morale Scale (PGCMS), which consists of three components: non-agitation, life satisfaction, and aging satisfaction. For each dimension, the items were collapsed into two manifest mean score indicators [14]. Internal consistency was good, as indicated by Cronbach’s Alphas of .82, .58, and .68 for non-agitation, life satisfaction, and aging satisfaction, respectively.

Educational level was self-reported. Those who reported that they had a degree (i.e., from a university or technical college) were considered to have a higher education.

Multimorbidity was assessed with a list of 24 chronic conditions based on the Charlson Comorbidity Index, which has been used in other surveys as well [15, 16]. Participants were given the opportunity to name up to two more conditions. Those who reported five or more separate conditions were considered multimorbid.

### Statistical analysis

Latent class analysis (LCA) refers to modelling with categorical latent variables (classes) that represent subpopulations where population membership is not known but is inferred from the data [17]. The number of latent classes was chosen based on Akaike’s and Bayesian information criterion (AIC, BIC), where lower values indicate better trade-off between the complexity and the goodness of fit of the model. Further, the Lo-Rubin Test (LR), where a significant value indicates that the present model is superior compared to one with less classes was used. In a consecutive step, we applied structural equation modelling to regress current PA and satisfaction on manifest PA biography classes. PA class allocation, sex, age, and current PA were manifest indicators, whereas each dimension of life satisfaction was modelled as a latent factor with two manifest indicators (mean scores of items). Gender and age served as covariates in the model. Missing values were accounted for by applying the Full Information Maximum Likelihood procedure (FIML) using Mplus v7.3, which incorporates all available data into the model estimation, i.e., retaining cases for which missing data are present [18]. The FIML procedure typically yields unbiased estimates even if data is missing at random. Fully standardized coefficients were reported.

## Results

The sample had an *N* = 419 with a mean age of 66.5 years (*SD* = 4.9) and an age range of 60–95 years (Table 1). In all, 70% of respondents were married and 47% were women. On average, participants had a relatively high level of education (64.2% had a university degree or a degree from a technical college).

**Table 1:** Characteristics of the participating older adults by latent class

	Total^1^		Increasingly active	Consistently active	Consistently inactive	Decreasingly active
N (%)	419		147 (35)	103 (25)	75 (18)	94 (22)
Variable	M (SD)	Empirical scale range	M (SD)	M (SD)	M (SD)	M (SD)
Age	66.6 (5.0)	60.0–95.0	67.3 (31.7)	66.7 (31.9)	66.4 (14.3)	66.2 (20.7)
Gender, female	1.5 (0.5)	1.0–2.0	1.3 (0.2)	1.5 (0.3)	1.6 (0.3)	1.5 (0.3)
Higher education, n (%)	269 (64.2)	0.0–1.0	88 (59.9)	57 (55.3)	54 (72.0)	70 (74.5)
Multimorbidity^3^, n (%)	277 (66.1)	0.0–1.0	95 (64.6)	76 (73.8)	45 (60.0)	61 (64.9)
Current PA	0.6 (0.9)	0.0–5.5	0.7 (0.8)	0.7 (1.0)	0.5 (0.8)	0.4 (0.2)
PA biography						
Youth	4.4 (1.4)	0.0–1.0	3.3 (1.0)	5.4 (1.0)	3.4 (1.0)	5.0 (1.0)
30 years	3.9 (1.4)	0.0–1.0	2.8 (0.8)	5.3 (0.8)	2.4 (0.8)	4.4 (0.8)
50 years	3.9 (1.4)	0.0–1.0	3.9 (0.8)	5.4 (0.8)	2.2 (0.8)	3.7 (0.8)
60 years	4.2 (1.4)	0.0–1.0	4.7 (0.8)	5.3 (0.8)	2.9 (0.8)	3.9 (0.8)
Satisfaction						
Life satisfaction	4.4 (0.9)	1.5–5.3	4.4 (0.9)	4.6 (0.7)	4.1 (0.9)	4.2 (1.0)
Aging satisfaction	4.2 (0.9)	1.6–6.0	4.2 (0.9)	4.4 (0.8)	3.9 (1.0)	4.2 (0.9)
Non-agitation	4.0 (0.9)^2^	1.0–5.0	4.1 (0.8)^2^	4.2 (0.8)^2^	3.7 (1.0)^2^	3.9 (0.9)^2^

*Note. N* = 419. M = Mean. SD = Standard Deviation. PA = Physical activity

^1^ Total refers to values derived from the structural equation model. ^2^ Manifest mean values. ^3^ Five or more conditions; the empirical range of conditions was 0–12, which was coded 0–4 (multimorbidity absent) or 5–12 (multimorbidity present)

The model with four classes was the best fit, according to AIC and BIC values (Table 2). AIC and BIC dropped significantly for the model with 4 classes and made a less steep decline in further models with more classes. For the models with 2 (LR = 367.2, *p* < .001) and 4 (LR = 91.3, *p* < .01) classes, LR-Test was significant. Furthermore, the information criteria was lower in the model with 4 classes. Therefore, the 4-class model was chosen (Table 2).

**Table 2:** Characteristics of the participating older adults by latent class

Class	AIC	∆AIC	BIC	∆BIC	Adj LR	Entrophy
1	5412		5444			
2	5043	(369)	5095	(349)	367.17***	.81
3	4990	(53)	5063	(32)	60.67	.73
**4**	**4906**	**(84)**	**4998**	**(65)**	91.31**	**.75**
5	4880	(26)	4994	(4)	34.47	.79
6	4858	(22)	4991	(3)	31.45	.79

*Note.* AIC = Akaike’s Information Criterion, BIC=Bayesian Information Criterion. ∆ refers to the difference of an information criterion from a model with k classes compared with the information criterion from a model with k+1 classes. For AIC and BIC, lower values indicate better trade-off between the complexity and the goodness of fit of the model. LR = Lo-Rubin Test. Bold refers to the model that was chosen for further analyses. Levels of significance are ** *p* < .01, *** *p* <.001

**Fig. 1:**
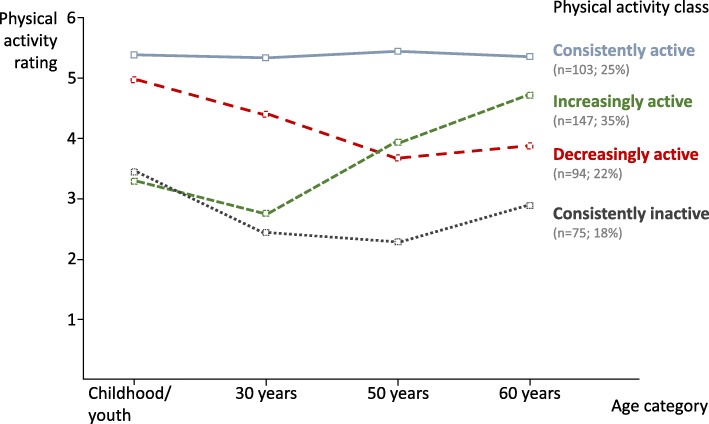
Latent classes of self-reported physical activity biographies

**Fig. 2:**
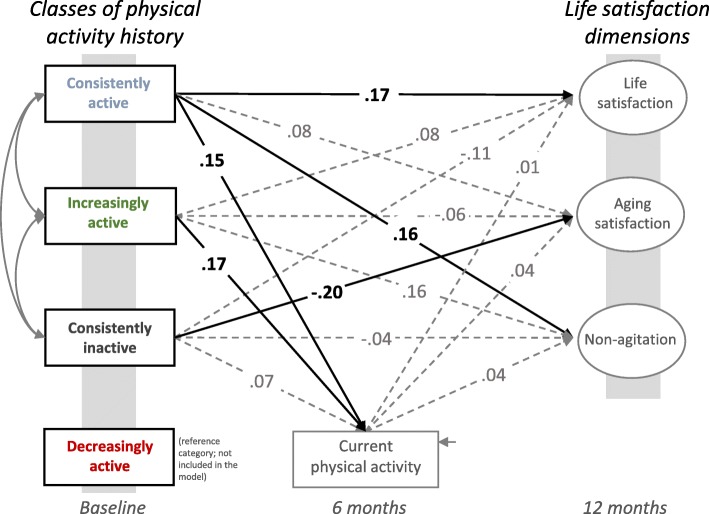
Longitudinal associations of physical activity biography classes with current physical activity and life and aging satisfaction. Fully standardized coefficients are presented. Numbers in bold represent significant coefficients on a *p* < .05 level. Age and gender covariates are not displayed

According to the PA biography patterns, participants were classified into four categories of physical activity: *increasingly active*, *consistently active*, *consistently inactive,* and *decreasingly active*. In 147 (35%) participants, PA levels increased over the lifespan, 103 (25%) reported to be consistently active over the lifespan, 75 (18%) participants were classified as consistently inactive, and 94 (22%) were decreasingly active throughout their life (see Fig. 1). In a consecutive step, the associations of PA biography classes with life and aging satisfaction when accounting for current PA were investigated. Results show that current PA was significantly related to being increasingly active (β = .17, *p* < .001) and consistently active (β = .15, *p* = .004; see Fig. 2). Only being in the consistently active class was indicative of aging satisfaction, non-agitation (β = .16, *p* = .033), and life satisfaction (β = .17, *p* = .028). Aging satisfaction (β = −.20, *p* < .001) was negatively related to being a member of the consistently inactive class. Gender (β = −.14, *p* = .020) was only related to life satisfaction while age (β = −.22, *p* = .005) was only associated with aging satisfaction. Current PA was not significantly related to any of the satisfaction indicators (all *p* > .05).

## Discussion

From this investigation of retrospectively reported PA biographies of older adults, four subgroups with different PA life course trajectories emerged from the data. Those older adults who had low amounts of PA in childhood and became increasingly active after the age of 30 were classified as *increasingly active*. Those who maintained high amounts of PA were classified as *consistently active*. Those who maintained low amounts of PA throughout the course of their life were classified as being *consistently inactive*. Finally, those who decreased their PA levels from childhood after age 30 were classified as *decreasingly active*. The consistently active classification was associated with current PA and life satisfaction; whereas being consistently inactive was negatively related to aging satisfaction, even when accounting for current PA. This research furthers our knowledge on the independent contribution of PA biographies to the variability in life and aging satisfaction in older adults. New insights include that there are types of older adults that became more active as they got older and consistently active individuals, which may be new indicators of successful aging [4].

The classes that emerged are in line with findings from other studies that compare an active class against more sedentary classes [7, 8]. In retrospective interviews, MacDonald et al. [7] found, as expected, that the number of activities and hours of PA for master athletes and sedentary older adults varied substantially across the lifespan. Likewise, we found older adults with active and inactive PA patterns; moreover, we found patterns that indicate that PA levels change throughout life. In the present study, consistent activity was positively associated with life satisfaction, which echoes literature of a positive current PA–life satisfaction relation in older adults [9]. Additionally, our data indicates that consistently low PA levels across the lifespan is negatively associated with aging satisfaction, which supports findings showing that PA continuity was adversely related to negative views on aging in women [19].

In our study, PA biographies explained variance in life and aging satisfaction, even when accounting for current PA levels. Independent from current levels of PA, being physically active throughout one’s life may be an independent resource for high life satisfaction in old age. Current PA was not independently related to life and aging satisfaction in the present study when accounting for patterns in PA biographies, which further underlines the significance of PA across lifespan. Concepts such as PA identity and PA habits, which may last over extended periods of life, can help explain why having an active lifestyle may be positively related to current PA and life satisfaction [20–22]. Moreover, Hirvensalo and Lintunen pointed out in their review of PA across the lifespan that concepts of a lifespan theory of PA such as tracking and transitions should be elaborated upon in PA research [20]. Tracking research highlights that early PA levels are related to PA levels in old age. Although PA patterns are relatively stable in old age, the authors argue that transitions such as widowhood and relocation into a specialized but less spacious facility such as a nursing home may be linked with reduced PA in old age [20].

### Strengths and limitations

This study has strengths and limitations. A strength of the study is the innovative approach to PA, which incorporates a perspective of current behavior and satisfaction with a genuine lifespan perspective using a simple and intuitive biography method. Other strengths include the longitudinal design and the sophisticated statistical analyses, wherein we combined LCA with structural equation modelling. One limitation is the validity of self-reported PA levels. However, the similarities in the results of this study with other studies on the same topic show the strength of the classes developed through this survey. We do not know whether people gave accurate reports of their lifespan PA levels. Retrospectively assessed PA biographies are not equivalent to actual PA across the lifespan. The results may thus be considered to be an individual’s mental representation of PA over their lifespan. While retrospectivly reported PA is related to actual PA, it can be considered a psychological concept in its own right. Our sample was relatively active and well educated, which limits the generalizability of the findings. Future studies should replicate the class structure using a more representative sample. We were not able to link the trajectories to common life events such as transition from primary to secondary school, transition from high school to college or university, marriage, becoming a parent, or retirement [23, 24]. Nor could we link the trajectories to non-normative critical life events such as the death of a spouse or the onset of a chronic disease such as rheumatoid arthritis [25]. These elements should be investigated in future studies.

### Implications for research, policy, and practice

While our findings have shown that specific PA biography patterns relate with life and aging satisfaction in older adults, future studies should investigate whether this pattern can be replicated in different settings and subgroups of older adults. Moreover, the relationship of PA biographies with indicators of physical functioning, including specific syndromes such as frailty, would be of interest for future studies. This was not included in our study, as we focused our investigation on aging satisfaction only. Finally, more research is needed that applies tailored interventional designs. It could be tested whether certain PA biographies (e.g., consistently inactive versus decreasingly active) are more responsive to information, which are framed to their PA background (e.g., cues that describe activity as pleasure or highlight the functional value versus cues that activating past habits and benefits of an active lifestyle [26]).

Implications for policy and practice include the importance of childhood fitness and PA for the entire life [20]. Although PA patterns across life appear to be stable for many individuals, tailored interventions that consider unique PA biographies may improve the effectiveness of PA promotion interventions for older adults [27–29]. Negative views on aging and aging satisfaction in individuals with low levels of PA across the lifespan may be target of interventions on an individual level as well as in pubic campaigns [10]. Results from a PA intervention study in older adults have shown that positive views on aging could be changed by interventions, which, in turn, result in changes in PA in that sample [30]. Self-reported PA biographies contribute valuable insight and enable researchers and health care professionals to gain a greater understanding of PA in older adults.

## Conclusions

This study identified distinct life course trajectories of physical activity in relation to life and aging satisfaction. Older adults who have been classified as consistently active were associated with being currently active, engaged in PA, and increased levels of life satisfaction. Consistently inactive individuals were less satisfied with their aging process. Whether interventions can be tailored to specific PA biographies needs investigation in future studies.

## Data Availability

On request (corresponding author).

## Abbreviations

AIC: Akaike’s Information Criterion
BIC: Bayesian Information Criterion
FIML: Full Information Maximum Likelihood
GPA: Grade Point Average
LCA: Latent Class Analysis
LR: Lo-Rubin
PA: Physical Activity
PGCMS: Philadelphia Geriatric Center Morale Scale
